# Multidisciplinary evidence of an isolated Neanderthal occupation in Abric del Pastor (Alcoi, Iberian Peninsula)

**DOI:** 10.1038/s41598-022-20200-z

**Published:** 2022-09-23

**Authors:** Santiago Sossa-Ríos, Alejandro Mayor, Cristo M. Hernández, Mariel Bencomo, Leopoldo Pérez, Bertila Galván, Carolina Mallol, Manuel Vaquero

**Affiliations:** 1 Departament de Prehistòria, Arqueologia i Història Antiga, Universitat de València, Avinguda Blasco Ibáñez 28, 46010 Valencia, Spain; 2 Àrea de Prehistòria; Departament de Prehistòria, Arqueologia, Història Antiga, Filologia Llatina i Filologia Grega, Facultat de Filosofia i Lletres, Universitat d’Alacant, Sant Vicent del Raspeig campus, Sant Vicent del Raspeig, 03690 Alicante, Spain; 3 Área de Prehistoria; Unidad de Docencia e Investigación de Prehistoria, Arqueología e Historia Antigua, Departamento de Geografía e Historia; Facultad de Humanidades, Universidad de La Laguna, Guajara campus, San Cristóbal de La Laguna, 38205 Santa Cruz de Tenerife, Spain; 4 Archaeological Micromorphology and Biomarkers Laboratory; Instituto Universitario de Bio-Orgánica Antonio González, Universidad de La Laguna, Anchieta campus, San Cristóbal de La Laguna, 38206 Santa Cruz de Tenerife, Spain; 5 Institut Català de Paleoecologia Humana i Evolució Social (IPHES-CERCA), Zona Educacional 4, Campus Sescelades URV (Edifici W3), 43007 Tarragona, Spain; 6 Departament d’Història i Història de l’Art, Universitat Rovira i Virgili, Avinguda de Catalunya 35, 43002 Tarragona, Spain

**Keywords:** Archaeology, Social evolution

## Abstract

Testing Neanderthal behavioural hypotheses requires a spatial–temporal resolution to the level of a human single occupation episode. Yet, most of the behavioural data on Neanderthals has been obtained from coarsely dated, time-averaged contexts affected by the archaeological palimpsest effect and a diversity of postdepositional processes. This implies that time-resolved Neanderthal behaviour remains largely unknown. In this study, we performed archaeostratigraphic analysis on stratigraphic units ive, ivf, ivg, va, vb and vc from Abric del Pastor (Alcoi, Iberian Peninsula). Further, we isolated the archaeological remains associated with the resulting archaeostratigraphic unit and applied raw material, technological, use-wear, archaeozoological and spatial analyses. Our results show a low-density accumulation of remains from flintknapping, flint tool-use and animal processing around a hearth. These data provide a time-resolved human dimension to previous high-resolution environmental and pyrotechnological data on the same hearth, representing the first comprehensive characterisation of a Neanderthal single occupation episode. Our integrated, multidisciplinary method also contributes to advance our understanding of archaeological record formation processes.

## Introduction

Recent research on Neanderthal settlement dynamics has revealed that their occupations were short-termed^1–3^ and the long-term campsite paradigm is no longer sustained^4^. The current focus is on isolating higher time-resolution units concealed in time-averaged archaeological deposits, but relatively little attention has been paid to the pursuit and characterisation of single occupation episodes as a means to approach Neanderthal behavioural variability^5–7^. Isolating single Neanderthal occupation events and exploring behavioural cues hidden in their material components and in their interrelationships may bring to light aspects, such as group mobility, technology or demography, relevant to advance ongoing debates on the Neanderthals, usually based on low-resolution temporal data.

A key challenge in approaching the single occupation episode is to assess the role of the different natural and anthropogenic processes leading to the formation of the archaeological record, since we still do not know to what extent these formation processes condition our inferences on behaviour^8^. For hunter-gatherer societies, the issue has been investigated in ethnoarchaeological studies, which take place at human narrow timescales. These allow for observations on the transformations of the material remains generated by specific human actions during single occupation episodes^8–11^. However, these minimal analytical units, which are the only to own this scale, are particularly difficult to be identified in Palaeolithic contexts because of the palimpsest effect, which implies an absence of examples^12^. Although time-averaged assemblages may be useful to recognise long-term trends, human behavioural variability is difficult to be assessed from mixed diachronic events. In the field of lithic technology, one of the best-known consequences of time-averaging is the complete reduction sequence fallacy, as pointed out by Dibble et al.^13^. This misconception could be also reflected in other analytical domains, such as hunting-related behaviour (i.e. the entire carcass transport fallacy) or palaeoenvironmental reconstruction (i.e. the mosaic landscape fallacy)^14,15^.

**Figure 1:**
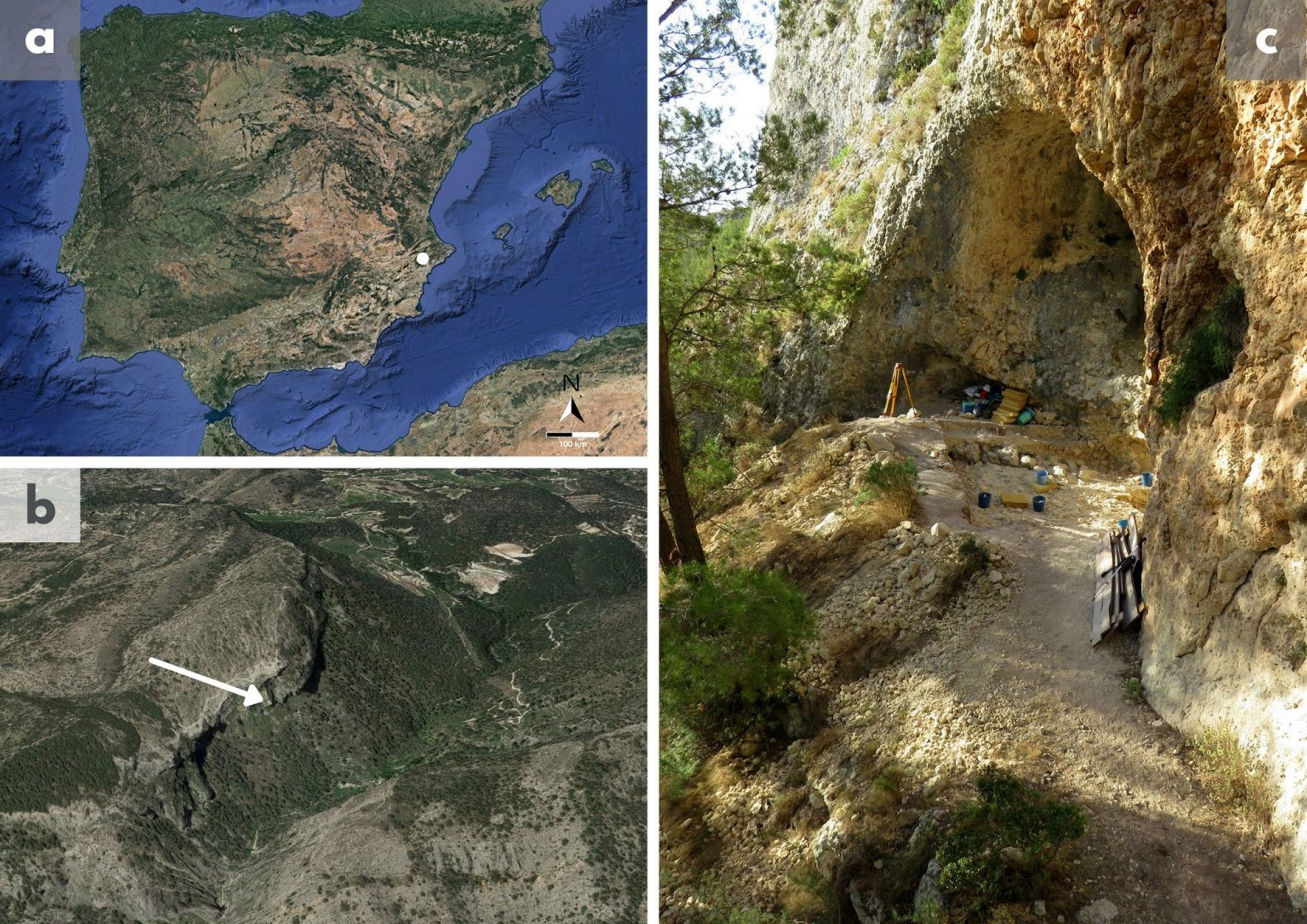
(**a**) Location of the Abric del Pastor site in the context of the Iberian Peninsula. The map was obtained with Google Earth Pro version 7.3 [Versiones de Earth—Google Earth]. (**b**) Location of the site in the context of the Mariola mountain range. (**c**) Site overview.

The Abric del Pastor site (Alcoi, Iberian Peninsula) (Fig. 1) is a small rockshelter with a Middle Palaeolithic archaeological deposit dated roughly between 63 ± 5ky BP and 48 ± 5ky BP and composed of granulometrically organised sandy, gravelly and clastic layers shaping a concave morphology^16–18^. The material record is integrated by low-density, stratigraphically discrete assemblages, whose features are favourable towards effective palimpsest dissection^16,17^. This is supported by the current excavation surface, which covers a major part of the rockshelter (40 of 60 m^2^).

Unit ivf is an example of this. It was investigated using a high-resolution interdisciplinary methodology that yielded palaeoenvironmental and pyrotechnological data, including the presence of a well preserved, simple, in situ, open hearth (i.e. H17), fueled with juniper wood^18^. The ash layer preserves molecular traces of herbivore fat^19^. These data show that this context is ideal for further investigations of behavioural aspects through a time-resolving approach.

Here, we present an interdisciplinary study that has allowed to recognise and characterise a low-density hearth-related accumulation representing a high-resolution human event within unit ivf. This study has combined archaeostratigraphic, raw-material, technological, use-wear, archaeozoological, and spatial analyses. Our integrated results, coupled with the previous data, become a chance of providing the features of a Neanderthal occupation episode.

## Results

### Archaeostratigraphy

**Figure 2:**
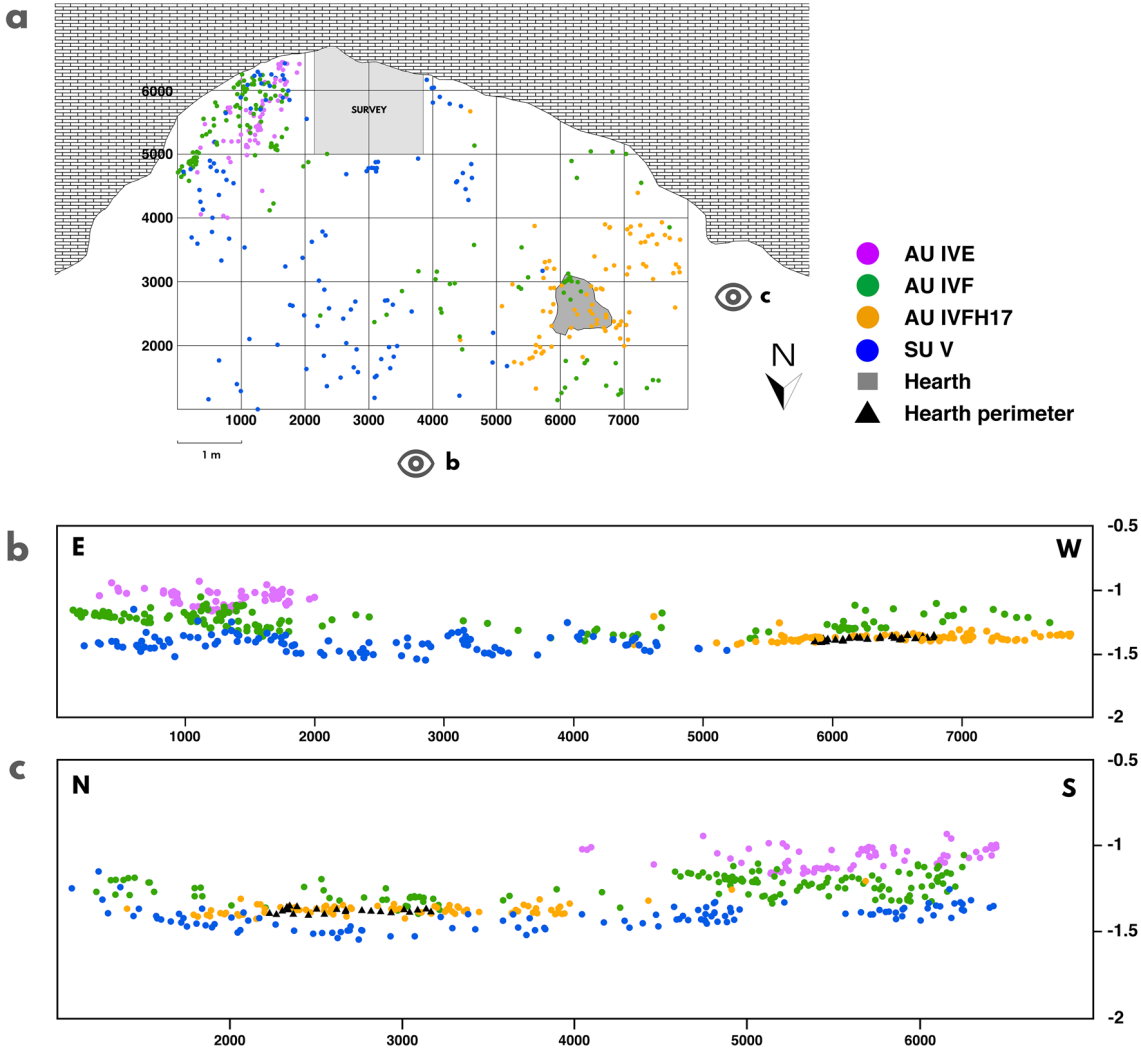
Representation of the archaeostratigraphic units (AU). (**a**) Horizontal plot. (**b**) Vertical plot (east–west). (**c**) Vertical plot (north–south).

The archaeostratigraphic analysis resulted in identification of three AUs: ive, ivf and ivfH17 (Fig. 2). From these, we are going to focus on the latter, which is partially located in the western part of the site and consists of 11 lithic products, 78 faunal remains and a combustion structure (i.e. H17). It has been defined by the following observations. The distinction between ivfH17 and ivf is better observed in the east–west general section, while its difference regarding SU v is more noticeable in the north–south general section, due to the natural slope of the SUs forming a slightly concave shape (Fig. 2). It should be noted that, in the western part of the site, no more remains have been recovered in the 5 cm below. This absence results in an archaeological gap beneath AU ivfH17.

**Figure 3:**
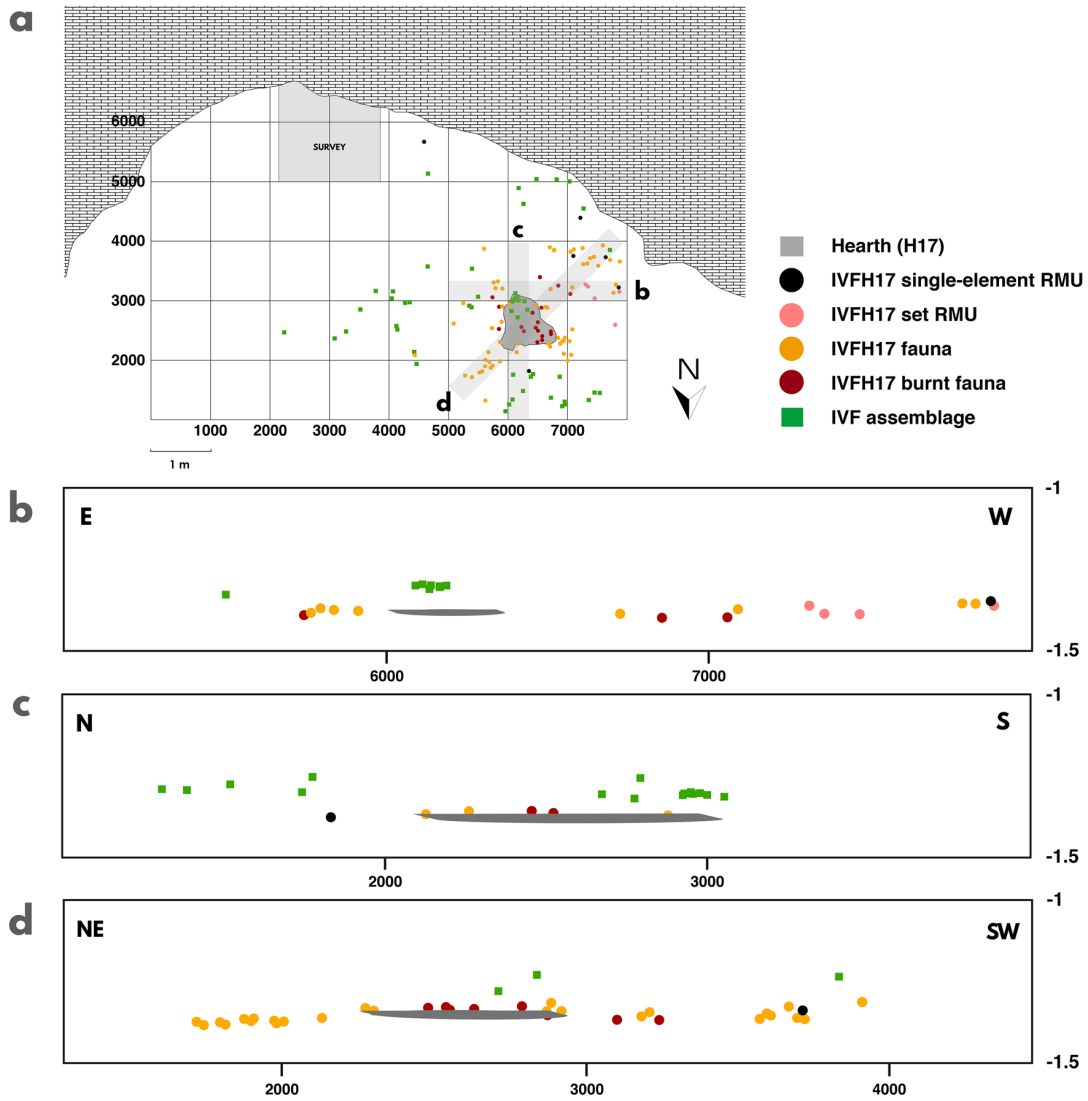
Archaeostratigraphic representation of the AU ivfH17 assemblage in comparison to the AU ivf assemblage. (**a**) Horizontal plot. (**b**) Cross-section (east–west). (**c**) Cross-section (north–south). (**d**) Cross-section (northeast-southwest).

In particular, the 40 cm-wide cross-sections were oriented to illustrate the separation of AU ivfH17 from ivf (Fig. 3a). The vertical distinction between AU ivfH17 and SU v is observed in the general north–south section (Fig. 2c). We plotted the assemblages from ivf and ivfH17, and distinguished, in the second case, the record components: the hearth surface, the partially refitted set raw material unit (RMU), the single-element RMUs, and the faunal bones, both heated and unburnt. On the one hand, the east–west cross-section shows the vertical connection between the set RMU and the faunal assemblage, as well as the vertical difference with regards to the upper assemblage (i.e. minimal vertical distance: 6 cm) (Fig. 3b). On the other hand, the north–south and the northeast-southwest cross-sections display the vertical contact among the faunal assemblage, particularly those burnt bones, and the hearth perimeter, as well as the vertical difference between ivfH17 and ivf assemblages (i.e. minimal vertical distance: 5 cm) (Fig. 3c, d).

### Raw materials

**Figure 4:**
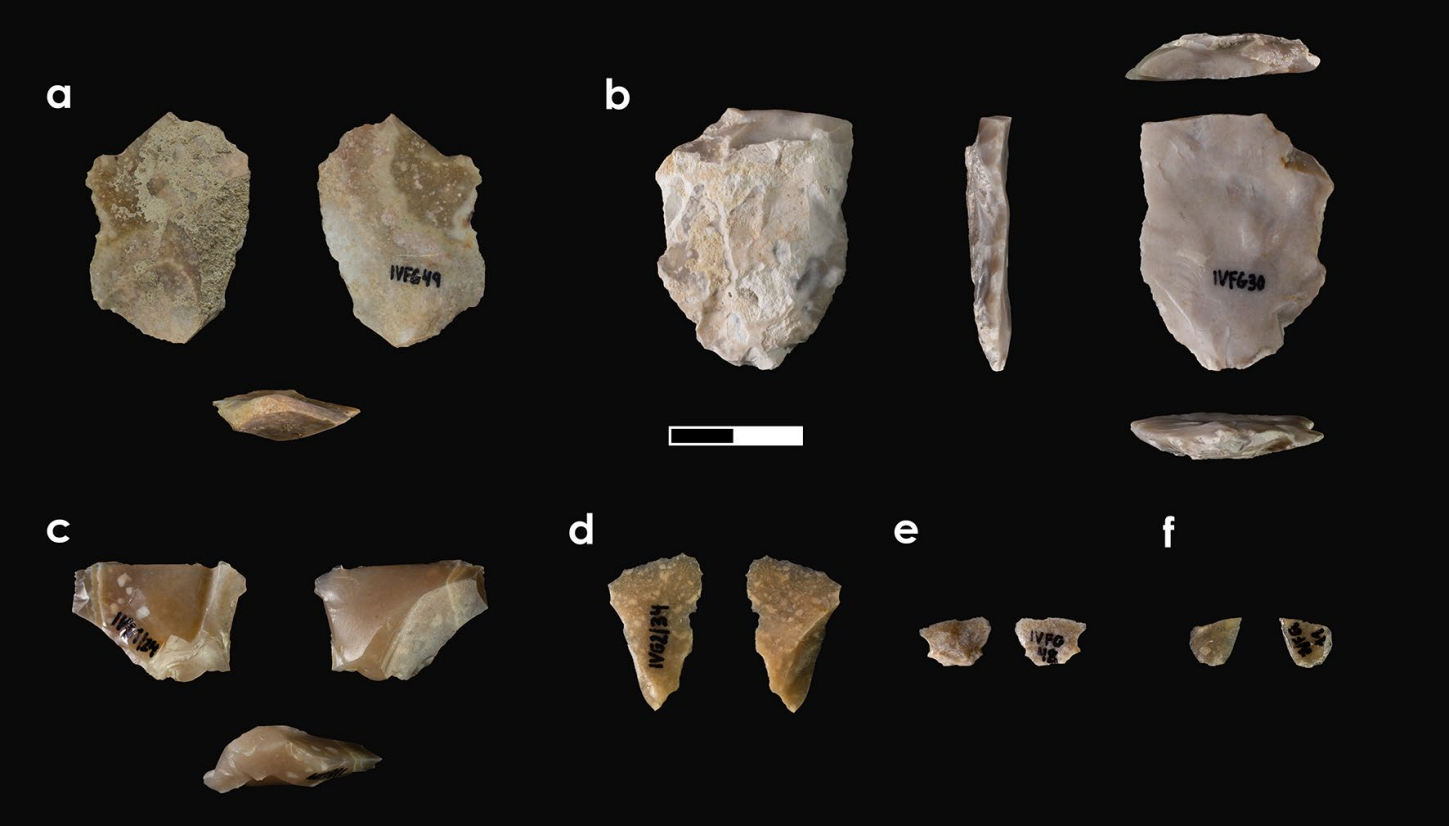
Single-element RMUs.

**Figure 5:**
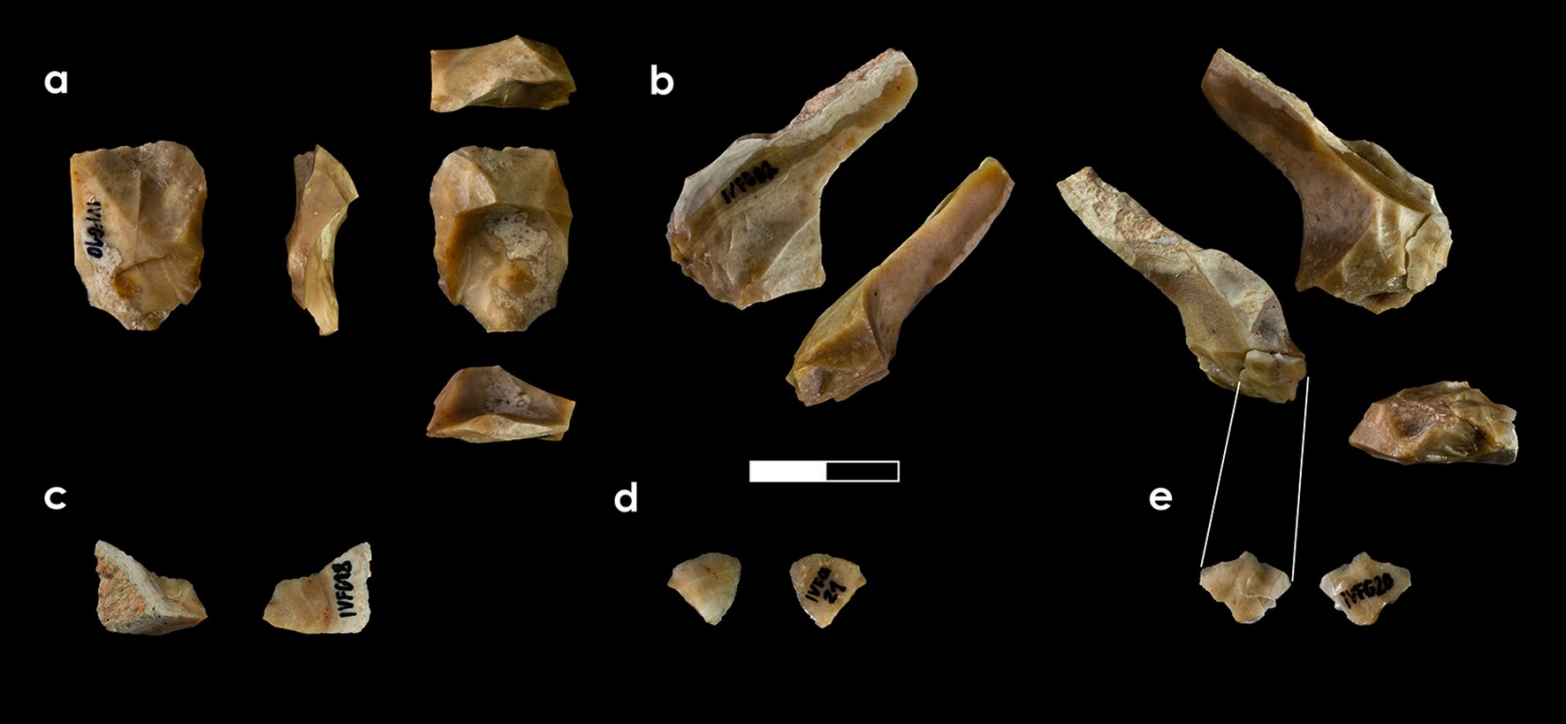
Set RMU. The reffitting connection is indicated by parallel lines.

The 11 lithic products are made on previously characterised flint types^20,21^: nine on Mariola flint and two on Serreta flint (Table S2; Figs. 4, 5). The Mariola assemblage is composed of four single-element RMUs and one set RMU comprising five artefacts. It shows different signs of natural postgenetic alterations linked to polishing and abrasion occurring after its release from bedrock and prior to its gathering. One product displays slight colour change from anthropogenic thermal alteration (i.e. yellow to pink) and small scales. No postgenetic features were observed on the Serreta flint objects, which were grouped into two single-element RMUs.

### Technology

The lithic assemblage is composed of 11 flakes (Table S3; Figs. 4, 5), five of which belong to the set RMU, and six are single-element RMUs. These latter are: one Mariola non-cortical centripetal flake (Fig. 4a), one Mariola cortical retouched flake (Fig. 4b), one Serreta *débordante*-like centripetal flake (Fig. 4c), and three small flakes, two on Mariola and one on Serreta flint (Fig. 4d–f). These six artefacts correspond to six different knapping sequences displaying distinct phases (Table S3).

The Mariola set RMU, here onwards ivf.M1, is composed of a surpassed flake showing centripetal core features (Fig. 5a), a flake with a cortical back (Fig. 5b), and three small flakes (Fig. 5c–e). The cortical flake, which is also the largest one, displays a series of small, flat, unipolar removals in the proximal area that led to bulb and butt removal, as well as dorsal face modification. One of these removals has been refitted with one of the three small flakes mentioned above (ivf.M1-R1; Fig. 5b,e). In the opposite side of the large flake, there is a rebounded fracture affecting the ventral face and departing from the distal area. This fracture, together with the above mentioned characteristics, are common features among splintered pieces (i.e. *pièces esquillées*)^22^, so its complete characterisation will be given in the next section being that these features have not been considered technical, but use-related (vid. use-wear for further information).

RMU ivf.M1 represents an operational chain on a Mariola flint nodule. The elements within it are representative of different stages of a Levallois recurrent centripetal knapping sequence (Table S3). This statement is supported by the features of the surpassed flake (Fig. 5a), which preserves the hierarchical edge between both striking and flaking platforms. It also shows several dorsal negatives characterised by a centripetal direction and a peripheral polarity. Furthermore, the technical features of the small flakes are along the same lines (Fig. 5c,d; Table S3), excepting the refitted one.

### Use-wear

Six elements (54.5%) were analysed for use-wear traces, which were identified in four of them related to the work of semihard/hard materials, and animal materials (Table S4).

**Figure 6:**
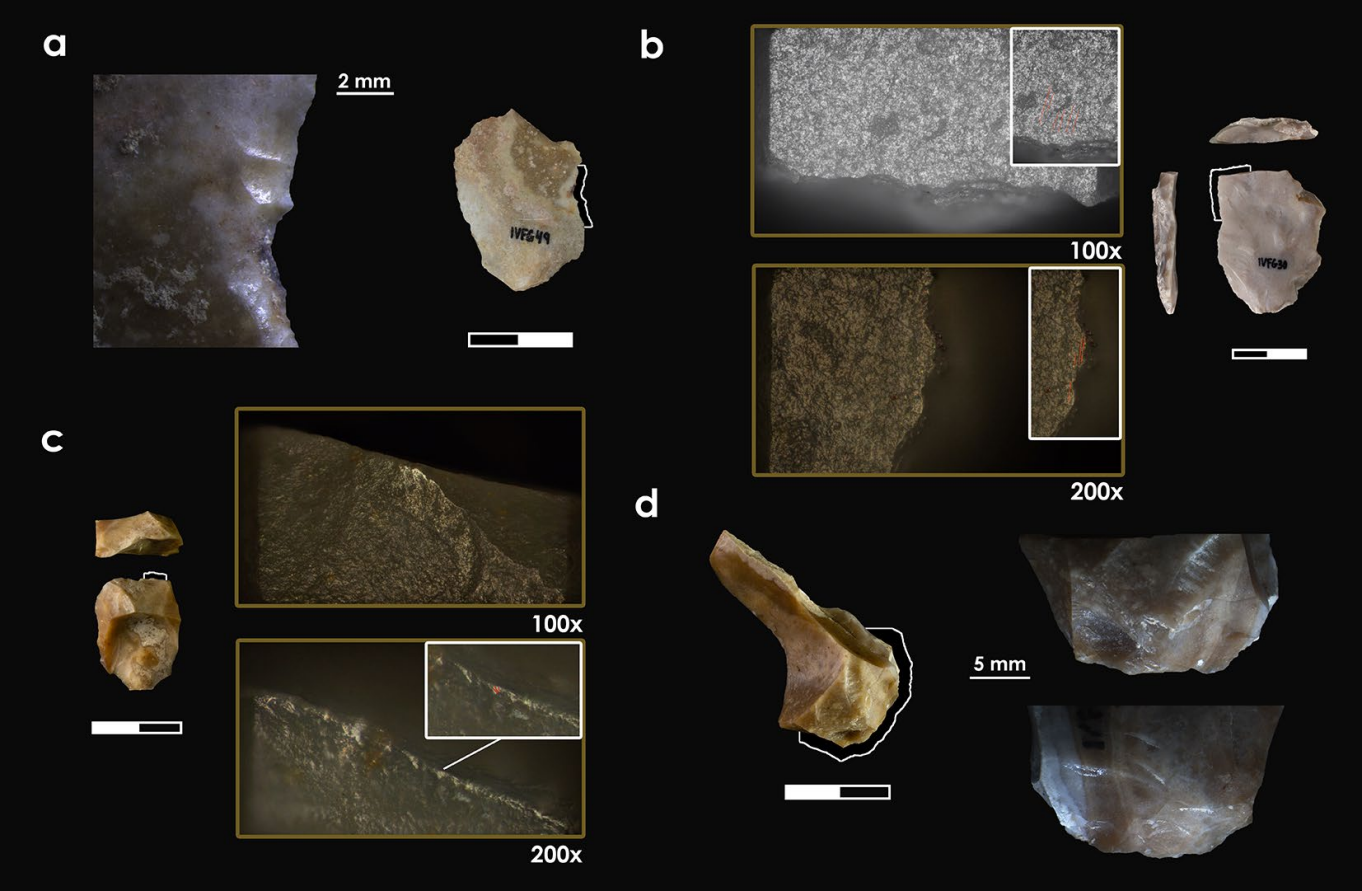
Tools displaying use-wear traces and detailed photos.

Concerning the single-element RMUs, we report two artefacts displaying use-wear. One is the Mariola centripetal flake, which has traces of working on a hard or semihard material. In this case, thermal alteration only allowed us to characterise the scars, which are large, semicircular, both aligned and superposed, and exhibit a feather termination (Fig. 6a). Its distribution on one side of the edge and its perpendicular orientation indicate a transversal motion related to a scraping action. The second one is the Mariola retouched flake, which shows traces of cutting of animal tissue (Fig. 6b). The flake edge is rounded, with long striations, and at the apex, there is a dull, rough-textured, domed polished exhibiting medium-large scars. These are, both aligned and superposed, with feather and step terminations associated with well-developed polished spots in the highest parts of the topography which indicates a contact with hard material (e.g. cartilage, bone). The distribution of the polish, scars and striations on both sides of the edge suggest a longitudinal motion, related to a cutting action.

RMU ivf.M1 contains two elements with use-wear traces. One is linked to bone cutting and exhibits a very bright polish, parallelly aligned to the edge, with a smooth texture and a flat topography (Fig. 6c). The scars are large, quadrangular and with step terminations, along with medium-sized, semicircular and feather terminations. The presence of short and deep striations with a parallel orientation on the edge, as well as the distribution of polishing on both sides of the edge, indicate a longitudinal movement of the tool related to a cutting action. The second one displays large, superposed, quadrangular and irregular scars, with a step termination and with a perpendicular orientation to the edge (Fig. 6d). These features, together with previously described technological features, suggest that the flake represents a splintered tool associated with percussion activity on hard material.

### Archaeozoology

The faunal assemblage is composed of 78 bone fragments, among which we have been able to recognise two species (*Cervus elaphus*, n2; *Capra pyrenaica*, n1) and one subfamily (*Caprinae*, n2). These remains represent at least three individuals (Table 1). However, this minimum number of individuals can be reduced to two, if we base ourselves on the fact that anatomical parts identified as belonging to the *Caprinae* subfamily are not repeated amongst the elements determined as *Capra pyrenaica*. The taxonomically undetermined bones were classified by weight categories. The medium-size group predominates (n56: 70.88%), followed by indeterminate (n15: 18.98%) and small-size remains (n2: 2.53%). Since medium and small sizes in SU iv are represented only by red deer and wild goat, respectively^23^, we assume that they correlate with these taxa.

**Table 1:** Archaeozoological data from the ivfH17 assemblage.

Taxa	Element	NR	Cut marks	Thermal alterations	Weathering	Erosion	Concreteness	Corrosion	Manganese	Sediment pressure	Roots marks
Caprinae	Metapodial	2									1
*Capra pyrenaica*	Tibia	1					1		1		1
*Cervus elaphus*	Metapodial	1					1		1	1	
	First phalanx	1					1				
Medium size	Vertebra	1			1				1		
	Thoracic vertebra	1			1				1		
	Ribs	3		1			2	1	2		2
	Ulna	1					1				
	Splinter	21	1	3	2		14	3	8		2
	Diaphysis	25	4	5	2		22	2	20		8
Small size	Ribs	1					1		1		
	Diaphysis	1		1		2	1				
Indeterminate	Splinter	16		3			21		9		
	Cancellous bone	3		5			3				

There is a predominance of postcranial remains, of which almost all are appendicular. The taxonomically classified remains are a red deer (*Cervus elaphus*) metapodial and a first phalanx, a wild goat (*Capra pyrenaica*) tibia and two *Caprinae* metapodials*.* In the medium-size group, skeletal representation is dominated by diaphysis fragments, splinters and ribs (Table 1). The small-size group is integrated by a diaphysis and rib fragment. Lastly, we report several splinters and cancellous bone fragments in the indeterminate group.

The entire assemblage shows a high degree of fragmentation (97.46%), of which 68.35% was old fresh-fractured, mostly into the medium-size group. Additional anthropogenic modifications include thermal alteration (24.05%) and cut-marks (6.33%). Burnt bones exhibit different degrees of heating, mostly charring (i.e. black surfaces), followed by mild heating (i.e. from brown to black) and near calcination (i.e. from black to grey). Cut-marks were identified on four diaphysis fragments and a splinter belonging to a medium-sized individual. No predator damage alterations, such as carnivores or raptors were observed. The degree of postdepositional alteration is high, and numerous bone surfaces display secondary carbonate and manganese oxide precipitation, corrosion, weathering, sedimentary pressure or root-marks.

### Spatial analysis results

**Figure 7:**
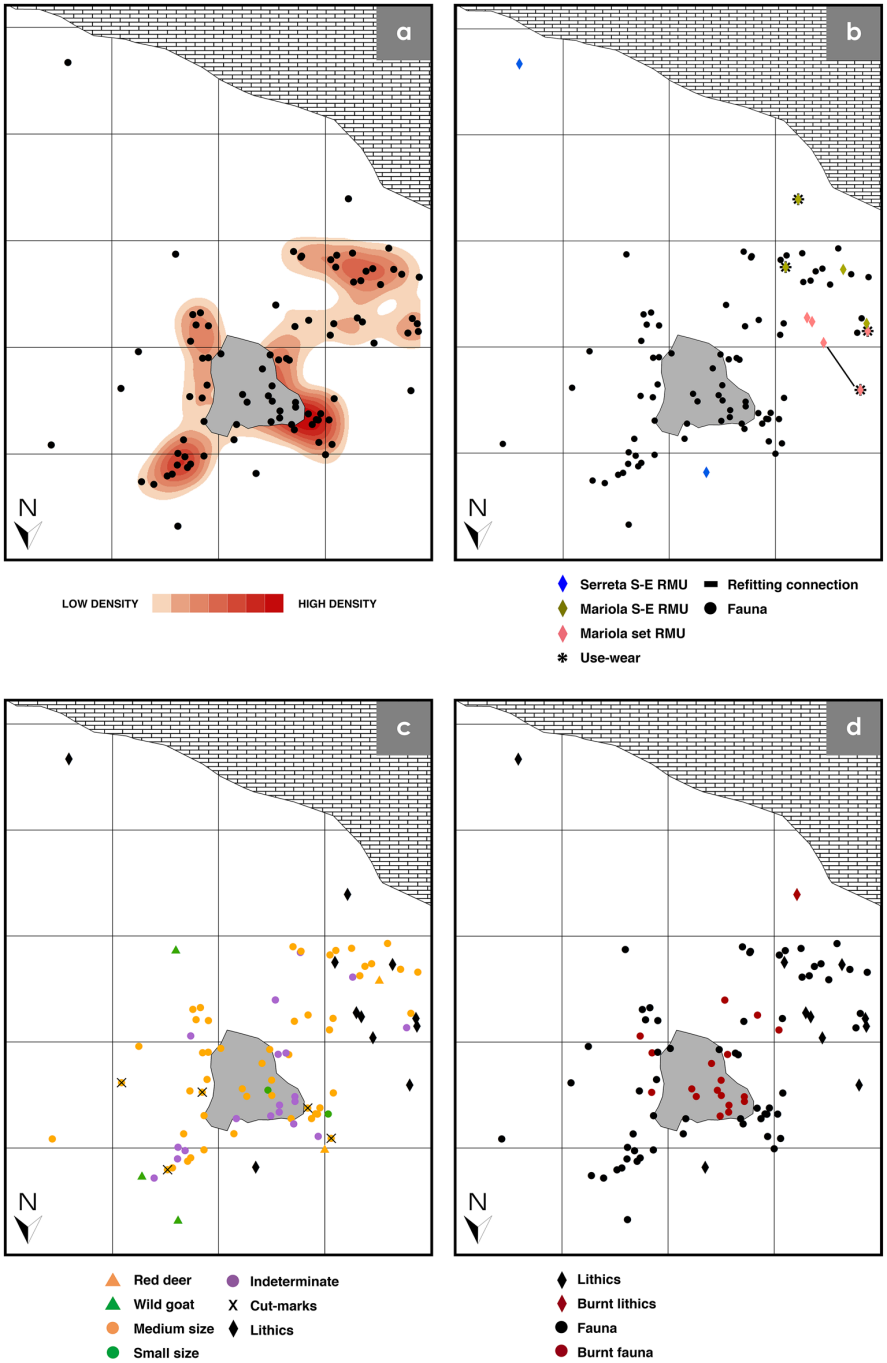
Horizontal plots of the AU ivfH17 assemblage used for spatial analysis. All the plots were generated with ArcGIS Desktop ArcMap version 10.5.1 [Esri Support ArcMap 10.5 (10.5.1)] (**a**) Kernel density map. (**b**) Lithic elements by RMU, flint type, use-wear traces and refitting connection. Single-element RMUs are abbreviated as S-E. (**c**) Faunal assemblage by taxa, size and cut-marks. (**d**) Burnt elements.

Spatially, the AU ivfH17 assemblage is distributed over ca. 20 m^2^ and constrained to the western part of the rockshelter (Fig. 7). The kernel density map displays two high-density spots adjacent to the hearth in the northern area, and another medium to high density spot in the southwest, which is 1.14 m from the hearth perimeter (Fig. 7a). The Serreta single-element RMUs are completely excluded from the spots. The average nearest neighbour (ANN) analysis applied to this group confirms its dispersion (ratio: 1.8941). For the rest of the assemblage, the ANN shows clustering ratios (Table S5).

Specifically, the lithic assemblage (Fig. 7b) is clustered in the western area with no remains inside the hearth. The Mariola set RMU is in the west of the fire, with a 0.56 m distance between the two refitted elements. The four Mariola single-element RMUs are found in the southwest area. In this group, the heated flake is 1.96 m away from the hearth centre and 1.59 m from the hearth perimeter (Fig. 7d). The two Serreta single-element RMUs are separated from the rest of the lithic assemblage. One of them is in the northern area and the other one is in the southeast.

Regarding the used tools (Fig. 7b), the retouched flake associated with butchering activity is 0.98 m to the southwest of the hearth perimeter. It is surrounded by bone fragments without cut-marks, including those belonging to *Cervus elaphus* and to medium-sized animals. The thermoaltered centripetal flake used to work on a hard or semihard material has a similar position (1.59 m to the southwest of the hearth perimeter). Nevertheless, there is a material gap of 0.5 m-radius around this piece. In the case of the set RMU, the flake used to work on a hard material is 1.26 m to the southwest of the hearth perimeter, while the splintered tool is 1.09 m west of it. Considering a 0.5 m radius for each case, the former artefact is more strongly related to the deer and medium-sized animal remains in the southwest. Regarding the distance from the nearest bone with cut-marks to the two used tools, the splintered piece is 0.89 m away from it., whereas the other one is 1.27 m.

The faunal assemblage is located around the hearth, with a minor distribution in the eastern area (Fig. 7c). The assemblage located inside the hearth (n16) is composed of medium-sized taxa (n9: 56.25%), indeterminate remains (n6: 37.50%) and small-sized taxa (n1: 6.25%). The assemblage located outside the fire (n62) shows predominance of medium-sized taxa (n43: 69.35%), followed by indeterminate (n13: 20.97%), *Cervus elaphus* (n2: 3.23%), *Caprinae* (n2: 3.23%), *Capra pyrenaica* (n1: 1.61%), and small-sized taxa (n1: 1.59%). The burnt remains are mostly situated inside the hearth (n11: 61.11%), but some are also found also outside of it (n7: 38.89%) (Fig. 7d). Finally, the bone fragments with cut-marks are distributed in the north of the assemblage, near to the hearth perimeter. Two of them are further than 0.5 m from the hearth perimeter, whereas the other three are closer than 0.5 m.

## Discussion

The integrated, interdisciplinary data presented in this paper offer the possibility of isolating and characterising human high-resolution events.

Archaeostratigraphic dissection reveals a vertically discrete assemblage of archaeological remains (lithic, faunal and combustion remains) framed between AU ivf at the top and SU v at the base. This deposit has been labelled AU ivfH17. This unit is composed of a hearth (H17) in the west, and different anthropogenic materials around it: 11 flint remains, both on Mariola and Serreta types, four of them showing use-wear traces, and 78 bone remains of red deer and wild goat.

The postgenetic alterations observed on the Mariola flint objects indicate fluvial source procurement, more specifically the upper course of the Serpis river (~ 3–5 km southeast)^20,21^. Even though Serreta flint could be gathered in the same contexts, we did not observe postgenetic alterations to support this possibility. The observed technological features reveal fragmentation of the operational chain even in high resolution contexts^24,25^. We identified the presence of elements that were introduced, used and abandoned, and others that were introduced, modified and taken out. These features allow considering cores and unretouched flakes as mobile toolkits.

In this sense, the bone assemblage shows a highly fragmentary input of appendicular and axial parts of red deer (n2) and wild goat (n5). Most of the cut-marks and burnt elements are related to the single red deer individual, possibly indicating direct consumption on site. The presence of herbivore fat lipid residues in the hearth sediment supports this hypothesis^19^. Use-wear analysis on lithics suggests low-intensity activities related to animal exploitation (i.e. butchery activity and bone processing). The use-wear traces observed on the splintered piece could be associated either to bone or to wood processing^26^. However, the exclusive use of the other tools on animal tissue and the high fragmentation degree of the faunal assemblage points to a bone processing activity, perhaps related to marrow extraction^27^.

Spatially, the ANN concentrated ratios observed in the entire assemblage and in almost all the material groups indicate a low postdepositional impact, as well as a short connection distance of the refitted set^28^. The distribution of remains clustered around the fire suggest the existence of a hearth-related assemblage^29–33^. The activities performed around H17 hearth (butchering, bone processing) are represented, on the one hand, by the bulk of the deer remains and the used tools in the southwest area, and on the other hand, by the cut-marked deer remains in the northern area.

Even isolating this short assemblage in the vertical deposit, we cannot assume synchrony in the horizontal plane. In this sense, there are some indications of possible diachrony amongst the H17 hearth-related assemblage inferred from taphonomic, anthropogenic and spatial features of: (1) the wild goat bones; (2) the burnt Mariola flint flake, and (3) the two isolated Serreta flint flakes.

First, the absence of anthropogenic modifications on the wild goat bones, might indicate a different moment and/or agent of deposition. Second, the burnt Mariola flint flake presents two distinguishing features from the rest of the assemblage. One is that thermal alteration affects the use-wear traces, indicating that it was used, subsequently heated and then discarded at the southern area of the rockshelter. The other feature is the postdepositional calcium carbonate cement on its surface, not observed on any other lithic object. Finally, the dispersed character of the wild goat assemblage, the burnt Mariola flake and the only two Serreta flints differs from the rest of the assemblage. Cross-checking this information with the clustered groups and their characteristics enables us to propose that diachrony exists between both assemblages: the main accumulation would be linked to a single moment, whereas these scattered materials might be the result of another or more depositional events.

Thus, excluding the two Serreta single-element RMUs, together with the wild goat remains and the burnt Mariola flake, we interpret the resulting assemblage as a potential single occupation episode. This event involved an input, use, transformation and abandonment/output of four Mariola RMUs and the processing-consumption of appendicular and axial parts of a red deer around the fire. The presence of animal fat in the hearth sediment is indirect evidence of deer meat consumption and/or tossing of meat in the fire.

The integrated, interdisciplinary method applied in this study allowed achieving the optimal spatiotemporal resolution that permits us to read Neanderthal behaviours. Regarding group mobility, the input record displays a movement from the southeast (~ 3–5 km), where the Mariola flint was gathered and deer hunting was more feasible due to ecological conditions^18,20,21^. Absence of Beniaia flint, which is a more distant flint type (~ 25–30 km) present in other AUs within Abric del Pastor^17^, strengthens the nearby character of the movements in this case. In this study, we also show that the difficulty in considering cores and unretouched flakes as mobile toolkits is a consequence of low resolution approaches. In the lithic assemblages affected by palimpsest, features interpreted as mobility indicators have been only recognised in some retouched single tools or in allochthonous raw materials^9,34–37^. Therefore, identifying mobile toolkits needs high-resolution contexts in order to observe the potential heterogeneity of technofunctional and raw material features.

We have brought to light partial evidence of use of a single flint toolkit (i.e. presence of a retouched tool and ten unretouched flakes), as well as indirect evidence of exported cores (i.e. absence of flint cores and on-site knapping). In parallel, there is evidence of transport to the rockshelter and on site butchery and consumption of selected anatomical parts of medium-sized prey. Such transportation patterns have been documented ethnoarchaeologically (e.g. Hazda populations)^38,39^, but not verified in Pleistocene archaeological contexts due to their low degree of spatiotemporal resolution^40,41^. Likewise, our time-resolved use-wear data indicates relationships between lithic and bone records linked to an animal-processing activity. This connection is scarcely observable in time-averaged contexts^6^.

By narrowing down the time scale of the archaeological framework, we have identified a possible single Neanderthal occupation episode representing a brief stay in a rockshelter. Our results raise the possibility that the small size of the archaeological assemblage, made up of few lithic and bone remains and a single simple hearth, its fragmented character and its spatial distribution might be representative of a Neanderthal single occupation episode. Are record formation processes in larger assemblages produced by a sum of single occupation events such as the one represented by AU ivfH17? This possibility could be investigated in Neanderthal contexts comprising time-averaged dense palimpsests, such as caves and rockshelters, but also in open-air sites where the anthropogenic deposits are vertically separated but the horizontal diachrony must be tested^42^. Approaches aimed at palimpsest dissection allow us to identify the elementary units of which the archaeological assemblages are composed and, therefore, to assess a behavioural variability that can be hidden in the assemblage-as-a-whole inferences.

## Materials and methods

In this work, we have taken into account the three-dimensional georeferences of 67 lithic elements, 356 faunal remains and 1 combustion structure belonging to SUs ive, ivf, ivg, va, vb and vc for archaeostratigraphic analysis (Table S1). After the vertical dissection had been carried out, we have focused on one from those higher-resolution analytical frameworks that have been obtained: AU ivfH17. This new unit comprises 11 flint remains and 78 faunal bones and a simple hearth. The lithic and faunal assemblage have been analysed through raw material, technological, use-wear, archaeozoological and taphonomic methodologies. Ultimately, we integrated the record information from this new analytical framework for consolidating the archaeostratigraphic dissection and achieving spatial distribution patterns (see Supplementary Information for methodological details).

### Site overview

The Abric del Pastor site is located in Alcoi (Alacant, eastern Iberian Peninsula). It is a rockshelter situated at 820 m above sea level, on an escarpment at the eastern foothills of Mariola mountain range. It is specifically found on the right bank of El Cint ravine, which connects to the upper and middle courses of the Benissaidó river. Geomorphologically, the rockshelter is a partially eroded karstic tube formed during the Miocene on alternating beds of Cretaceous dolomitic limestone and Serravallian Miocene limestone cobble conglomerates^43^. Erosion of the karstic tube resulting in the rockshelter occurred during the Pleistocene and is connected with incision and phreatic activity of the Benissaidó river^16^.

The site has a potential occupation surface area of 60m^2^, of which 40m^2^ are included in the excavation surface. The stratigraphic sequence is 1.5 m-thick and has been subdivided into six units: i-vi from top to base. (Fig. S2) (see Supplementary Information for site details). SU ivf (Fig. S1) is a gravitational gravelly-sandy deposit derived from roof spall. It was excavated during the 2016 and 2017 archaeological field seasons. Previous studies of SU ivf have provided chronometric OSL data indicating that this unit dates roughly to 63 ± 5ky BP^18^. Anthracological, archaeozoological (i.e. microfaunal and macrofaunal), biomolecular and micromorphological data available from SU ivd (overlying ivf) indicate supramediterranean semiarid-cool environmental conditions^23^.

## Supplementary Information

Supplementary Information.

## Data Availability

All data produced and used in this paper are available under reasonable request to the corresponding author.
