# Supplementary material for: Multidisciplinary evidence of an isolated Neanderthal occupation in Abric del Pastor (Alcoi, Iberian Peninsula)
Source: Sci Rep. 2022 Sep 23;12:15883. doi: 10.1038/s41598-022-20200-z (PMC9508120; doi:10.1038/s41598-022-20200-z)
Supplement: Supplementary file 1 — Supplementary Information. [file 41598_2022_20200_MOESM1_ESM.pdf]

Supplementary Information

for

Multidisciplinary evidence of an isolated Neanderthal occupation in Abric del Pastor (Alcoi, Iberian peninsula)

Santiago Sossa-Ríos[*,1], Alejandro Mayor[2], Cristo M. Hernández[3,4], Mariel Bencomo[1], Leopoldo Pérez[1,5], Bertila Galván[3], Carolina Mallol[3,4], Manuel Vaquero[5,6]

[*] SSR is the corresponding author: sosrios@uv.es

[1] Departament de Prehistòria, Arqueologia i Història Antiga, Universitat de València. Avinguda Blasco Ibáñez 28, 46010 Valencia, Spain.

[2] Àrea de Prehistòria; Departament de Prehistòria, Arqueologia, Història Antiga, Filologia Llatina i Filologia Grega; Facultat de Filosofia i Lletres, Universitat d'Alacant (Sant Vicent del Raspeig campus, Sant Vicent del Raspeig 03690 – Alacant, Spain)

[3] Área de Prehistoria; Unidad de Docencia e Investigación de Prehistoria, Arqueología e Historia Antigua; Departamento de Geografía e Historia; Facultad de Humanidades, Universidad de La Laguna (Guajara campus, San Cristóbal de La Laguna 38205 – Santa Cruz de Tenerife, Spain)

[4] Archaeological Micromorphology and Biomarkers Laboratory; Instituto Universitario de Bio-Orgánica Antonio González, Universidad de La Laguna (Anchieta campus, San Cristóbal de La Laguna 38206 – Santa Cruz de Tenerife, Spain)

[5] Institut Català de Paleoecologia Humana i Evolució Social (IPHES-CERCA), Zona Educacional 4, Campus Sescelades URV (Edifici W3), 43007 Tarragona, Spain.

[6] Universitat Rovira i Virgili, Departament d'Història i Història de l'Art, Avinguda de Catalunya 35, 43002 Tarragona, Spain.

Contents

- Extended site background
- Extended methods
- Extended data

Extended site background

The Abric del Pastor is located within the Serra de Mariola natural park, specifically in an area belonging to the municipality of Alcoi, headtown of L'Alcoià county, in the Valencian province of Alacant. The first archaeological excavation was performed by Mario Brotons in 1952 and 1953, exposing the stratigraphic units (SUs) I, II and III [1]. After this, it has been excavated yearly and sometimes even twice a year from 2005 to 2021, except for 2020. The 1.5m-thick stratigraphic sequence comprises six SUs with three absolute dates (48±5ky BP in IVb, 63±5ky BP in IVd and 62±12ky BP in VI) [2] (Fig. S2):

SU I is a Holocene deposit with a very high content of caprine faecal matter, whereas SUs II, III, IV, V and VI correspond to the Late Pleistocene deposit identified to date and contain a smaller or larger density of Neanderthal material record.

Excepting SU II, which is a dark-brownish sedimentary relict truncated by SU I, the Late Pleistocene deposit formation process is based on the fall of the limestone conglomerate in different size ranges (i.e. blocks, clasts, gravels and sands), due to gravitational effects probably caused by gelifraction or other weathering effects occurred during cold and dry climatic periods [2]. SUs IV and V have been additionally subdivided into other SUs responding to distinguishable episodes of roof-fall and the consequent alternation of fine and coarse granulometric standards occurring postdepositionally (i.e. blocks and clasts, gravels and sands): IVa to IVg and Va to Vc, which is currently under excavation.

Fig. S1. a) excavation surface of SU IVf. b) H17 hearth on field. c) excavation surface of SU IVf with the H17 hearth.

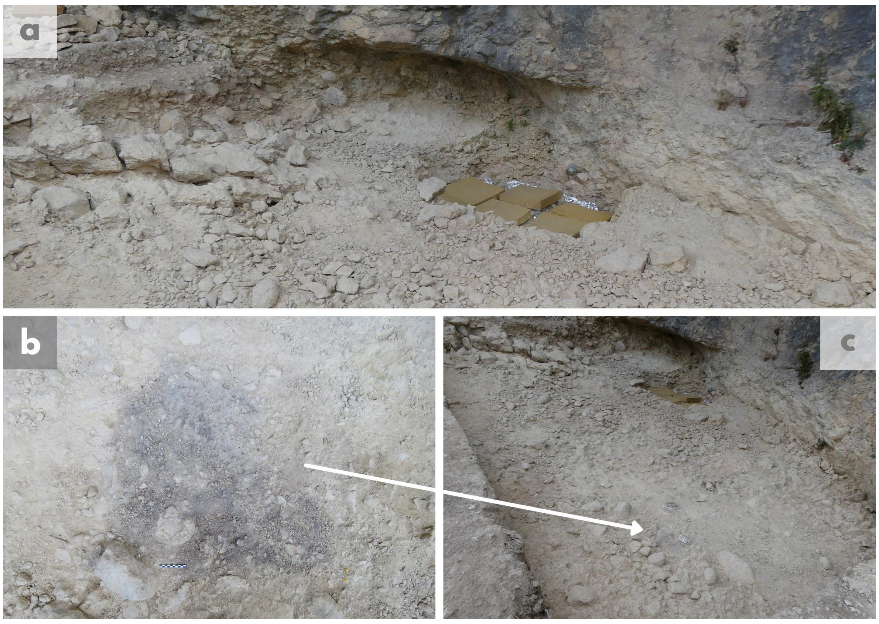

Fig. S2. Stratigraphic sequence of Abric del Pastor (modified from Mallol et al. [2]).

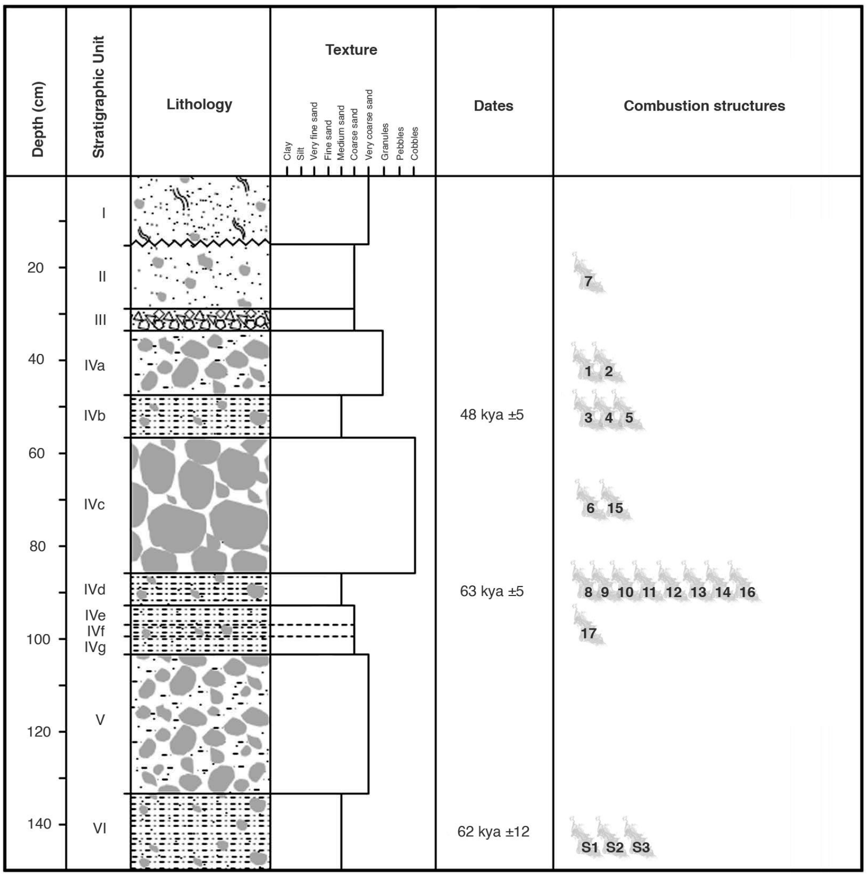

Extended methods

Archaeostratigraphic and spatial analyses

All the assemblage has been georeferenced on field using both a Sokkia® iM-50 Series and a Leica Geosystems® FlexLine TS-02 total stations. Afterwards, vertical cross-sections and horizontal plotting have been generated using Environmental Systems Research Institute® (ESRI) ArcGIS Desktop ArcMap version 10.5. Three-dimensional observation and correlation of archaeological materials have been made using ESRI® ArcGIS Desktop ArcScene version 10.5.

Three-dimensional data (X, Y, Z) has served for establishing new analytical units through palimpsest dissection, focusing on vertical material gaps and relationships between the archaeological record features (e.g. burnt bones and hearth perimeter) [3,4]. One of these units individualised through archaeostratigraphic analysis has been taken here (i.e. the hearth-related assemblage associated with H17) to be studied in depth. The archaeological record belonging to this one comprises 11 flint artefacts, 78 faunal remains and 1 hearth. Two-dimensional georeferences (X-Y) regarding this assemblage, including the hearth, have been utilised in order to observe scattering or concentration dynamics on the surface and spots of high frequency of materials [5-7]. For this, we have applied the Kernel density analysis and the Average Nearest Neighbour using ESRI® ArcGIS Desktop ArcMap version 10.5.

Raw material analysis

Here, we have performed a three-step analysis in order to recognise geogenic and postgenetic features, and to individualise RMUs within the assemblage of the above-mentioned 11 flint elements.

Both macroscopic and microscopic observations have been carried out in order to achieve the identification of flint types following Molina et al. [8]. For doing this, we take into account the translucence degree (i.e. translucent or opaque), the grain size (i.e. fine, medium or coarse), the internal structure (i.e. microcrystalline, cryptocrystalline or opaline), the presence or absence of internal fissures and recrystallisations, the type and quantity of inclusions (i.e. geodes, sands or bioclasts), the cortex features (i.e. texture, colour and thickness), the thickness of

halos within subcortical and endocortical areas, and the broad colour spectrum. The microscopic analysis was developed using a Euromex® DZ Series binocular loupe between 0.8 and 80 magnifications.

We have carried through also the postgenetic approach defined by Fernandes and Raynal [9], which was utilised by Molina et al. [8] and Mayor et al. [10] for the Serpis riverbed, from the upper course, subsidiary watercourses and mountainous surroundings to the seashore. In this way, it is possible to recognise potential provisioning areas visited by Neanderthal groups through the signs of physical and chemical alterations occurring on flint (e.g. crashing, polishing, abrasion, round-shaping, patination, permeation). An extensive representation of these posgenetic alterations for the Serpis riverbed context is in Mayor et al. [10] Supplementary Material.

Additionally, we used this information (i.e. geogenic and postgenetic data) for the identification of RMUs. We observed the assemblage in order to establish set RMUs (i.e. two or more flint elements belonging to the same original nodule) in order to address the anthropogenic input dynamics of siliceous raw material masses into the site [11-13]. In the same way, the elements that we could not associate with other pieces, are single-element RMUs since they equally represent single original nodules.

Technological analysis

Morphometric and technical features of the 11 lithic remains were studied by the next means:

- Measuring length for flakes and major axis for cores, width for flakes and minor axis for cores, thickness for both, and weight for all the products.
- Observing the main morphological characteristics of each flake (i.e. shape, section, butts, edge outlines and bulbs) and core (i.e. shape, section, striking platforms and debitage surfaces), and the cortical percentages.
- Reading the diacritic schemes of flakes and cores, as a tool for diachronically ordering and qualitatively characterising the technical actions performed within the knapping sequences (i.e. number and direction of negatives, superpositions, degree of invasiveness or marginality and surface hierarchy).

Furthermore, we aimed to look for refits through the whole assemblage, since the refitted sequences allow us to better understand technical strategies and technological conceptions utilised for exploitation [14,15].

Use-wear analysis

Use-wear analysis was developed on 6 of the 11 lithic remains. The other 5 pieces were not analysed due to their reduced size. The observation of use-wear traces was compared with references [16-21] and with an experimental program (Fig. S3) for a better interpretation.

The 6 lithic tools were analysed under the Leica M165C stereo microscope (3.65x-60x) and the Leica DM6000M metallographic microscope (50x-500x). Before the analysis, the tools were cleaned with warm water and neutral soap (Hygenia, Magnum Blue).

Fig. S3. Use-wear traces from the experimental program. a) Traces after 7 minutes cutting bone (100x). b) Traces after 7 minutes cutting bone (200x). c) Traces after 75 minutes working on butchery activity (meat and bone). d) Traces after 20 minutes working on butchery activity (skin and meat).

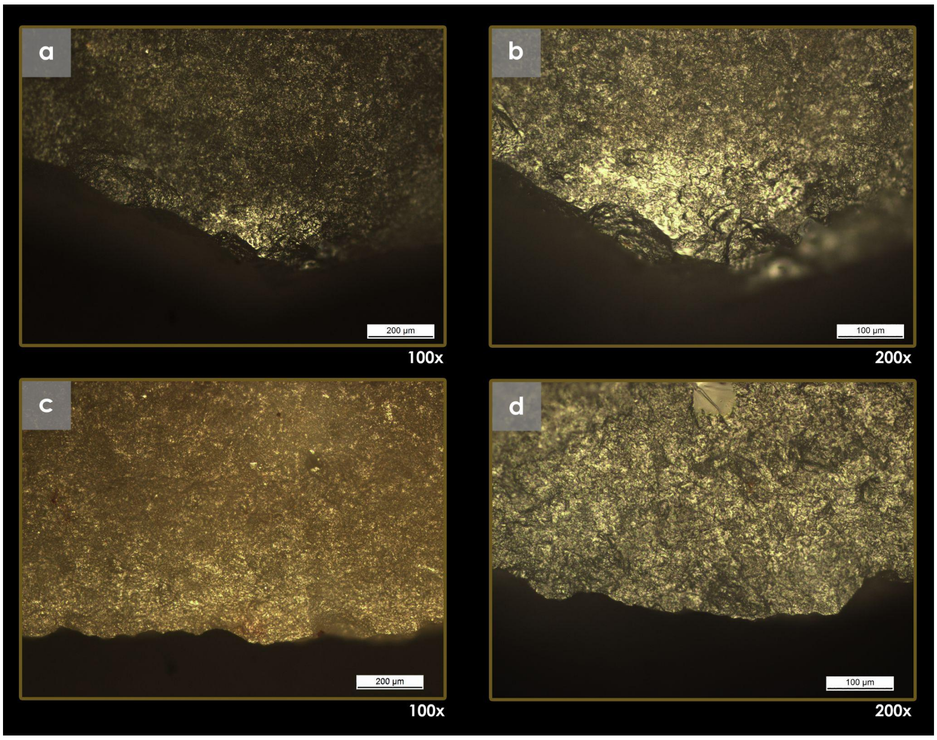

Archaeozoological and taphonomic analyses

Archaeozoological analysis was performed on the 78 faunal remains using standard methods [22,23]. All faunal remains were taxonomically and anatomically identified, except for bone fragments without anatomical characters or a high degree of taphonomic modifications. Non-identified specimens were classified into long bone, flat bone or articular and associated with a weight-size category based on bone density, circumference and the thickness of the cortical surface: large-sized (>300kg), medium-sized (100-300kg), small-sized (5-<100kg) and very small-sized (<5kg) [24,25]. The abundance measures employed are reduced to the number of remains (NR), number of identified specimens (NISP), the minimal number of elements (MNE) and the minimal number of individuals (MNI), due to the taphonomic conditions of the sample.

All remains were analysed under microscope Leica M165C stereo light. All dimensions (length, width, thickness) were measured for each bone fragment and the fracture analysis followed the criteria established by Villa and Mahieu [26] and the morphotypes created by Real et al. [27]. Bone surface modifications were observed and quantified to identify damage caused by anthropogenic activity (thermal alteration, percussion and butchering marks) or predator damage (tooth marks, digestion), as well as the diverse diagenetic processes that produce alterations on bone surfaces (erosion, sediment concreteness, roots marks, weathering, pigmentation, trampling) [28-34].

Supplementary data

Table S1. Total remains and combustion structures from the SUs studied in this work.

Stratigraphic unit	Lithic remains	Faunal remains	Combustion structures
IVe	5	46	
IVf	6	92	1 (H17)
IVg	11	134	
Va	0	16	
Vb	11	17	
Vc	34	52	
**Total**	**67**	**357**	**1**

Table S2. Main raw material features of the assemblage.

Flint type	Flint type representation (%)		Total/cortical elements	Proportion (%)	Number of elements by approximate percentage of cortical surface (%)					Cortex type	Number of elements with signs of postgenetic processes
	By number of elements	By raw material units			>0 - 25	>25 - 50	>50 - 75	>75 - <100	100	Neocortex	Rolling stigmata
Mariola	81.81	71.43	9/3	33.33	1	0	1	1	0	3	3
Serreta	18.19	28.57	2/0	0.00	0	0	0	0	0	0	0
**Total**	**100**	**100**	**11/3**	**27.27**	**1**	**0**	**1**	**1**	**0**	**3**	**3**

Table S3. Main technological features of the assemblage.

Raw material unit	Nomenclature in Fig. 4 and 5	Flint type	Refitting set	Size	Weight (g)	Butt	Flaking-face blanks	Striking polarity	Flaking direction	Technical phase	Retouch following Laplace [35]
Single element (Fig. 4)	a	Mariola	-	Small	4.09	Faceted	4	Peripheral	Centripetal	Full production	-
	b	Mariola	-	Medium	7.72	Point-shaped	2	Undiagnosed	Undiagnosed	Decortication	lat.dex.[Smd cconv]
	c	Serreta	-	Very small	1.99	Nonexistent	6	Peripheral	Centripetal	Convexity recovering	-
	d	Mariola	-	Very small	0.27	Flat	3	Undiagnosed	Unidirectional	Core reconfiguration	-
	e	Mariola	-	$<10mm^2$	0.09	Line-shaped	3	Undiagnosed	Unidirectional	Core reconfiguration	-
	f	Serreta	-	$<10mm^2$	-	Line-shaped	Undetermined	Undiagnosed	Unidirectional	Core or flake reconfiguration	-
Set RMU IVf.M1 (Fig. 5)	a	Mariola	-	Very small	2.77	Flat	4	Peripheral	Centripetal	Full production	-
	b	Mariola	IVf.M1-R1	Small	6.28	Removed	Undetermined	Undiagnosed	Undiagnosed	Full production	-
	c	Mariola	-	Very small	0.44	Flat	1	Undiagnosed	Unidirectional	Decortication	-
	d	Mariola	-	$<10mm^2$	0.16	Flat	-	Undiagnosed	Unidirectional	Core or flake reconfiguration	-
	e	Mariola	IVf.M1-R1	$<10mm^2$	0.15	Flat	-	Undiagnosed	Undiagnosed	Tool use	-

Table S4. Use-wear characterisation of the tools presenting traces.

Nomenclature in Fig. 6	Tool morphology	Edge morphology	Worked material	Action
a	Rectangular	Concave	Semi-hard/hard material	Scraping
b	Rectangular	Apex	Animal tissue	Cutting
c	Rectangular	Straight	Bone	Cutting
d	Irregular	Convex	Hard material	Percussion

Table S5. Average Nearest Neighbour analysis results from the total assemblage and the different material groups. Single-element RMUs are abbreviated as S-E RMUs.

Group	Expected mean distance	Observed mean distance	Ratio	*p*-value
AU IVfH17	0.3352	0.1538	0.4590	0,0001
Mariola set RMU	1.7320	0.2670	0.1541	0.0002
Mariola S-E RMUs	1.5811	0.5730	0.3624	0.0147
Serreta S-E RMUs	2.2360	4.2354	1.8941	0.0155
Deer	0.4303	0.1445	0.3359	0.0001
Wild goat	1.4142	0.7765	0.5491	0.0537

References

1. Galván, B. Hernández, C. M., Francisco, M. A., Molina, F. J. & Tarriño, A. La producción lítica del Abric del Pastor (Alcoy, Alicante). Un ejemplo de variabilidad musteriense. *Revista Tabona*, **17**, 11-61.
2. Mallol, C. *et al*. Fire and brief human occupations in Iberia during MIS 4: evidence from Abric del Pastor (Alcoy, Spain). *Sci*. *Rep*. **9**, 18281; 10.1038/s41598-019-54305-9 (2019).
3. Machado, J., Hernández, C. M., Mallol, C. & Galván, B. Lithic production, site formation and Middle Palaeolithic palimpsest analysis: in search of human occupation episodes at Abric del Pastor Stratigraphic Unit IV (Alicante, Spain). *J*. *Archaeol*. *Sci*. **40**, 2254-2273; 10.1016/j.jas.2013.01.002 (2013).
4. Mora, R. Roy, M., Martínez, J., Benito, A. & Samper, S. Inside the palimpsest: identifying short occupations in the 497D level of Cova Gran (Iberia) in *Short-term occupations in Paleolithic archaeology* (eds. Cascalheira, J. & Picin, A.) 39-69 (Springer, 2020).
5. Sánchez-Romero, L. *et al*. New insights for understanding spatial patterning and formation processes of the Neanderthal occupation in the Amalda I cave (Gipuzkoa, Spain). *Sci*. *Rep*. **10**, 8733; 10.1038/s41598-020-65364-8 (2020).
6. Spagnolo, V. *et al*. Neandertal camps and hyena dens: living floor 150A at Grotta dei Santi (Monte Argentario, Tuscany, Italy). *J*. *Archaeol*. *Sci*. *Rep*. **30**, 102249; 10.1016/j.jasrep.2020.102249 (2020).
7. Hammond, H., Zilio, L., Peralta, S. & Moreno, J. E. Intra-site spatial analysis of lithic assemblage and refitting of an open-air site in a lacustrine landscape from central Patagonia. *J*. *Archaeol*. *Sci*. *Rep*. **42**: 103367; 10.1016/j.jasrep.2022.103367 (2022).
8. Molina, F. J., Tarriño, A., Galván, B. & Hernández, C. M. El sílex del Prebético de Alicante: tipos, variabilidad y áreas de captación y talla del Pleistoceno. *Cuad*. *Prehist*. *Arqueol*. *Univ*. *Granada* **26**, 283-311 (2016).
9. Fernandes, P. & Raynal, J. P. Pétroarchéologie du silex: un retour aux sources. *Comptes Rendus Palevol* **5**: 829-837; 10.1016/j.crpv.2006.04.002 (2006).
10. Mayor, A. *et al.* An instance of Neanderthal mobility dynamics: a lithological approach to the flint assemblage from stratigraphic unit VIII of El Salt

rockshelter (Alcoi, eastern Iberia). *J. Archaeol. Sci. Rep.* **44**, 103544; 10.1016/j.jasrep.2022.103544 (2022).

11. Roebroeks, J. W. *From find scatters to early hominid behaviour: a study of Middle Palaeolithic riverside settlements at Maastricht-Belvédère (the Netherlands)* (Leiden University Press, 1988).
12. Vaquero, M. The history of stones: behavioural inferences and temporal resolution of an archaeological assemblage from the Middle Palaeolithic. *J. Archaeol. Sci.* **35**, 3178-3185; 10.1016/j.jas.2008.07.006 (2008).
13. Vaquero, M. Introduction: Neanderthal behavior and temporal resolution of archaeological assemblages in *High resolution archaeology and Neanderthal behavior: time and space in level J of Abric Romaní (Capellades, Spain)* (ed. Carbonell, E.) 1-16 (2012).
14. Cziesla, E. On refitting of stone artefacts in *The big puzzle: international symposium on refitting stone artefacts* (eds. Cziesla, E., Eickhoff, S., Arts, N. & Winters, D.) 9-44 (Holos, 1990).
15. Romagnoli, F. & Vaquero, M. The challenges of applying refitting analysis in the Palaeolithic archaeology of the twenty-first century: an actualised overview and future perspectives. *Archaeol. Anthropol. Sci.* **11**, 4387-4396; 10.1007/s12520-019-00888-3 (2019).
16. Tringham, R., Cooper, G., Odell, G., Voytek, B. & Whitman, A. Experimentation in the formation of edge damage: a new approach to lithic analysis. *J. Field Archaeol.* **1**, 171-196; 10.1179/jfa.1974.1.1-2.171 (1974).
17. Hayden, B. Snap, shatter, and superfractures: use-wear of Stone skin scrapers in *Lithic use-wear analysis* (ed. Hayden, B.) 207-229 (Academic Press, 1979).
18. Keeley, L. H. *Experimental determination of stone tool uses* (University of Chicago Press, 1980).
19. Vaughan, P. C. *Use-wear analysis of flaked stone tools* (University of Arizona Press, 1985).
20. Anderson-Gerfaud, P. Aspects of behavior in the Middle Palaeolithic: functional aspects of stone tools from southwest France in *The emergence of modern humans: an archaeological perspective* (ed. Mellars, P. A.) 303-326 (Cornell University Press, 1990).

21. González, J. E. & Ibáñez, J. J. The quantification of use-wear polish using image analysis: first results. *J. Archaeol. Sci.* **30**, 481-489; 10.1006/jasc.2002.0855 (1994).
22. Lyman, R. L. *Vertebrate taphonomy* (Cambridge Manuals in Archaeology, 1994).
23. Reitz, E. J. & Wing, E. S. (eds.) *Zooarchaeology* (Cambridge University Press, 2008).
24. Uerpmann, H. P. Animal bone finds and economic archaeology: a critical study of osteo-archaeological method. *World Archaeol.* 4, 307-322; 10.1080/00438243.1973.9979541 (1973).
25. Bunn, H. T. Patterns of skeletal representation and hominid subsistence activities at Olduvai Gorge, Tanzania, and Koobi Fora, Kenya. *J. Hum. Evol.* **15**, 673-690; 10.1016/S0047-2484(86)80004-5 (1986).
26. Villa, P. & Mahieu, É. Breakage patterns of human long bones. *J. Hum. Evol.* **21**, 27-48; 10.1016/0047-2484(91)90034-S (1991).
27. Real, C. *et al*. Abrigo de la Quebrada level IV (Valencia, Spain): interpreting a Middle Palaeolithic palimpsest from a zooarchaeological and lithic perspective. *J. Palaeolithic Archaeol.* **3**, 187-224; 10.1007/s41982-018-0012-z (2020).
28. Shipman, P. *Life history of a fossil: an introduction to taphonomy and paleoecology* (Harvard University Press, 1981).
29. Shipman, P. & Rose, J. Early hominid hunting, butchering, and carcass-processing behaviors: approaches to the fossil record. *J. Anthropol. Archaeol.* **2**, 57-98; 10.1016/0278-4165(83)90008-9 (1983).
30. Blasco, M. F. *Tafonomía y prehistoria: métodos y procedimientos de investigación* (Servicio de Publicaciones de la Universidad de Zaragoza, 1992).
31. Yravedra, J. Acumulaciones biológicas en yacimientos arqueológicos: Amalda VII y Esquilleu III-IV. *Trab. Prehist.* **63**, 55-78; 10.3989/tp.2006.v63.i2.17 (2006).
32. Domínguez, M. & Yravedra, J. Why are cut mark frequencies in archaeofaunal assemblages so variable? A multivariate analysis. *J. Archaeol. Sci.* **36**, 884-894; 10.1016/j.jas.2008.11.007 (2009).
33. Denys, C. & Patou-Mathis, M. *Manuel de taphonomie* (Errance, 2014).

34. Fernández-Jalvo, Y. & Andrews, P. *Atlas of taphonomic identification: 1001+ images of fossil and recent mammal bone modification* (Springer, 2016).
35. Laplace, G. *La typologie analytique et structurale: base rationnelle d'étude des industries lithiques et osseuses* (Centre National de la Recherche Scientifique, 1974).
